# Cadherins in the retinal pigment epithelium (RPE) revisited: P-cadherin is the highly dominant cadherin expressed in human and mouse RPE *in vivo*

**DOI:** 10.1371/journal.pone.0191279

**Published:** 2018-01-16

**Authors:** Xue Yang, Jin-Yong Chung, Usha Rai, Noriko Esumi

**Affiliations:** Wilmer Eye Institute, Johns Hopkins University School of Medicine, Baltimore, Maryland, United States of America; University of Florida, UNITED STATES

## Abstract

The retinal pigment epithelium (RPE) supports the health and function of retinal photoreceptors and is essential for normal vision. RPE cells are post-mitotic, terminally differentiated, and polarized epithelial cells. In pathological conditions, however, they lose their epithelial integrity, become dysfunctional, even dedifferentiate, and ultimately die. The integrity of epithelial cells is maintained, in part, by adherens junctions, which are composed of cadherin homodimers and p120-, β-, and α-catenins linking to actin filaments. While E-cadherin is the major cadherin for forming the epithelial phenotype in most epithelial cell types, it has been reported that cadherin expression in RPE cells is different from other epithelial cells based on results with cultured RPE cells. In this study, we revisited the expression of cadherins in the RPE to clarify their relative contribution by measuring the absolute quantity of cDNAs produced from mRNAs of three classical cadherins (E-, N-, and P-cadherins) in the RPE *in vivo*. We found that P-cadherin (CDH3) is highly dominant in both mouse and human RPE *in situ*. The degree of dominance of P-cadherin is surprisingly large, with mouse *Cdh3* and human *CDH3* accounting for 82–85% and 92–93% of the total of the three cadherin mRNAs, respectively. We confirmed the expression of P-cadherin protein at the cell-cell border of mouse RPE *in situ* by immunofluorescence. Furthermore, we found that oxidative stress induces dissociation of P-cadherin and β-catenin from the cell membrane and subsequent translocation of β-catenin into the nucleus, resulting in activation of the canonical Wnt/β-catenin pathway. This is the first report of absolute comparison of the expression of three cadherins in the RPE, and the results suggest that the physiological role of P-cadherin in the RPE needs to be reevaluated.

## Introduction

The retinal pigment epithelium (RPE), located between retinal photoreceptor cells and the choroid of the eye, is a single layer of pigmented epithelial cells with cobblestone-like morphology [1]. The RPE is essential for normal vision through multiple activities that support the health and function of retinal photoreceptors. The RPE constantly faces oxidative stress due to its large oxygen consumption and daily phagocytosis of photoreceptor outer segments, leading to accumulation of oxidative damage with age, which is thought to contribute to the loss of epithelial integrity and the development of diseases such as age-related macular degeneration (AMD) [1–3].

RPE cells are known to dedifferentiate and lose their fully matured state as a result of a variety of stresses, including oxidative stress and mechanical dissociation of cell-cell junctions [4–10]. Dissociation of cultured RPE cells leads to dedifferentiation of the cells into fibroblast-like cells through epithelial to mesenchymal transition (EMT) [5, 9]. EMT is a process in which cells lose cell-cell junctions and epithelial morphology and become fibroblast-like with increased mesenchymal markers [11–13]. RPE cells undergoing EMT contribute to scarring and wound contractions in proliferative vitreoretinopathy (PVR) as well as subretinal fibrosis in advanced AMD [14–16].

To maintain the integrity of epithelial cells, adherens junctions are critical by forming cell-cell contacts as protein complexes consisting of cadherin homodimers and p120-, β-, and α-catenins that link to actin filaments (F-actin) [17–19]. Cadherins are Ca^2+^-dependent cell adhesion molecules that connect neighboring cells through homophilic interaction of two homodimers on the cell surface [18, 20–22]. In most epithelial cell types, E-cadherin is the major cadherin responsible for forming and maintaining their epithelial phenotype [18, 20, 23]. However, it has been reported that RPE cells are different from other epithelial cells in terms of the major cadherin subtype that they express [24, 25].

Results of the expression of cadherin subtypes in the RPE have been conflicting. In cultured human RPE cells, N-cadherin rather than E-cadherin was dominantly expressed [25–27]. In the center of cultured porcine RPE sheets, where intact RPE cells were located, P-cadherin was abundantly detected, but it was lost at the edge of RPE sheets, where cells were migrating away and showed fibroblastic morphology with the expression of N-cadherin and vimentin [9]. *In situ* hybridization with mouse embryos showed that the outer layer (RPE) of the optic cup expressed N-cadherin until embryonic day 10.5 (E10.5) but switched to P-cadherin from E12 onward [28]. This study also showed that E-cadherin expression was not detectable in the RPE throughout embryonic and postnatal stages, indicating that each cadherin displays unique spatial and temporal expression patterns [28]. However, drawing general conclusions from these studies is challenging because they differ with regards to species (human, pig, and mouse), RPE source (cultured RPE cells, cultured RPE sheet, and *in situ* RPE), temporal stage (embryonic, postnatal, and adult), and methodology (Western blot, immunofluorescence, and *in situ* hybridization). In addition, these methods are not suitable for comparison of the expression levels across different cadherins.

The above-described findings suggest that a dominant cadherin subtype may be different between cultured RPE cells and mature RPE *in vivo*, and that P-cadherin may be the major cadherin in the latter that is more relevant to RPE physiology, but not in the former. However, this issue has not been clearly addressed so far. Importantly, mutations of human *CDH3* (the gene encoding P-cadherin) cause two rare autosomal recessive disorders: Hypotrichosis with Juvenile Macular Dystrophy (HJMD) [29–32] and Ectodermal Dysplasia, Ectrodactyly, and Macular Dystrophy (EEM syndrome) [33]. HJMD is characterized by early-onset hair loss with severe macular dystrophy, particularly at the RPE. Of note, P-cadherin-deficient mice are viable and fertile with no overt developmental abnormalities including ocular phenotype [34]. Besides the *in situ* hybridization analyses described above [28], to the best of our knowledge there is only one paper that analyzed P-cadherin in mouse RPE. Using immunofluorescence of P-cadherin, the authors reported that albino RPE cells were irregularly shaped with more loose and wider distribution of adherens junctions at the cell membrane than pigmented RPE cells, suggesting that the lack of melanogenesis may result in impaired RPE cell integrity in albino mice [35].

To address the unsolved issues of the role of cadherin subtypes in the RPE, in this study we revisited the expression of classical cadherins in RPE cells and determined their relative contribution by measuring the absolute cDNA quantity produced from mRNAs of E-, N-, and P-cadherins in human and mouse RPE *in situ*. Our results show that P-cadherin (CDH3) is the dominant cadherin in mature RPE cells. Using immunofluorescence, we confirmed that P-cadherin protein is localized at the cell-cell border of mouse RPE *in situ*. In addition, we also show that oxidative stress disrupts the localization of P-cadherin at cell-cell junctions at least temporarily, even when RPE cells look grossly intact, and that this oxidative stress-induced dissociation of adherens junctions leads to the translocation of β-catenin into the nucleus, a sign of activation of the canonical Wnt/β-catenin signaling pathway. This is the first report of absolute comparison of the expression levels of three classical cadherins in the RPE. Based on these results, we suggest that the physiological role of P-cadherin in the RPE needs to be reevaluated.

## Materials and methods

### Mice and sodium iodate (NaIO_3_) injection

All mice were treated in strict accordance with the recommendations in the Guide for the Care and Use of Laboratory Animals of the National Institutes of Health. The protocol was approved by the Johns Hopkins University Animal Care and Use Committee (Protocol Number: MO15M230). No procedures were performed on live mice, except tail vein injection. Euthanasia was performed with CO_2_ from a compressed gas cylinder followed by cervical dislocation, which is consistent with the American Veterinary Medical Association’s Guidelines for Euthanasia of Animals. Mice used in this study were 2–10 weeks old male C57BL/6J mice purchased (Jackson Laboratory, Bar Harbor, ME). To induce oxidative stress *in vivo*, mice (8–10 weeks old) were injected via tail veins with sodium iodate (NaIO_3_; S4077, MilliporeSigma, ‎St. Louis, MO) in phosphate-buffered saline (PBS) at the dose of 15 mg/kg body weight. Since NaIO_3_ is known to cause oxidative damage exclusively in the RPE, with no obvious systemic toxicity or histological damage in other tissues, it does not produce noticeable suffering or distress to mice.

### RNA preparation from human RPE

Total RNA of human RPE and retina was previously extracted directly from human donor eyes [36, 37]. Total RNAs of human RPE primary cells (named M1 as described below) and various human tissues were previously prepared and purchased, respectively for our prior studies [36, 38].

RPE primary cells were cultured following the published protocol [39]. Human donor eyes obtained from Eye Banks were dissected equatorially, and the cornea, lens, and retina were removed gently. The RPE cell layer was incubated with 2.4% dispase (Calbiochem, MilliporeSigma, Burlington, MA, USA) in Dulbecco’s Modified Eagle’s Medium (DMEM; Gibco, Thermo Fisher Scientific, Grand Island, NY) containing 100 mM sorbitol (MilliporeSigma) at 37°C for 45 min. The dispase solution was replaced with DMEM with 20% fetal bovine serum (FBS; Gibco, Thermo Fisher Scientific), and RPE cells were collected by gentle scraping. The RPE primary cells were maintained in 75 cm^2^ flasks coated with laminin (Upstate, MilliporeSigma) in DMED with 20% FBS and 50 μg/ml of Endothelial Cell Growth Supplement (ECGS; Upstate, MilliporeSigma). The RPE primary cultures became confluent in approximately 3 weeks, and the cells were split and subcultured in DMED with 10% FBS and 10 ng/ml basic FGF (bFGF; Upstate, MilliporeSigma). The medium was changed every 2 days. Among several RPE primary cell preparations, the one named M1 showed the best cobblestone-like morphology. For gene expression analyses, therefore, the third passaged M1 RPE cells were subcultured in DMED with 10% FBS and 10 ng/ml bFGF, and RNA was extracted using Trizol reagent (15596, Invitrogen, Thermo Fisher Scientific) 2 weeks after the cells reached tightly packed confluence (a total of 4 weeks in culture). At the time of RNA extraction, the M1 RPE cells exhibited a uniform cobble-stone-like appearance. We had analyzed the expression of RPE markers, *MITF*, *OTX2*, and *RPE65*, in these M1 cells in our prior studies [36]. The mRNA levels of *MITF* and *OTX2* in M1 RPE cells were similar to those in human RPE *in situ*; however, the mRNA level of *RPE65* was substantially lower (barely detected) in M1 RPE cells compared with that in human RPE *in situ* [36].

### RNA preparation from mouse RPE and choroid individually

To analyze gene expression in mouse RPE and choroid individually, we modified the RNA extraction method reported for only mouse RPE [40]. Briefly, mouse eyes were dissected to remove the cornea and lens, and the retina was peeled off to obtain the eyecup containing the RPE, choroid, and sclera. RPE cells were released by incubating the eyecup in 200 μl of RNAprotect cell reagent (76526, Qiagen, Valencia, CA) in a microcentrifuge tube at room temperature for 10 min followed by gentle tapping of the tube. Then, the choroid/sclera eyecup was transferred to a new tube containing 500 μl of Trizol, the released RPE cells were collected by centrifugation at 650 x *g* for 5 min at room temperature, and 500 μl of Trizol were added to the RPE cell pellets. The choroid/sclera eyecup and the RPE cell pellets were homogenized separately using a pestle grinder, and RNA was purified from each tissue by the two-step extraction strategy, i.e., first extracting RNA into the aqueous phase with Trizol and then purifying RNA from the aqueous phase with RNeasy Micro Kit (74004, Qiagen) following manufacturers’ instructions. To check cross-contamination, the mRNA levels of RPE markers (*Sox9*, *Otx2*, and *Rpe65*) and choroid markers (*Vwf* and *Col6a1*) were analyzed in RPE and choroid RNA samples by reverse transcription-quantitative PCR (RT-qPCR) using gene-specific primers (S1 Table).

### RT-qPCR

The mRNA levels of three mouse cadherin genes, *Cdh1* (the gene encoding E-cadherin), *Cdh2* (N-cadherin), and *Cdh3* (P-cadherin), in the RPE and choroid were analyzed by RT-qPCR. Total RNA from mouse RPE and choroid was prepared using the method described above. RT-qPCR was performed as previously described [41] with minor modifications. First-strand cDNA was synthesized from 200 ng of total RNA with random primers using SuperScript III reverse transcriptase (18080044, Invitrogen), and real-time PCR was performed with primers (S1 Table) and SYBR Green master mix using C1000 Thermal Cycler (Bio-Rad, Hercules, CA). Relative gene expression was calculated using the 2^−ΔΔCt^ method with *Gapdh*, *Hprt*, and *Actb* as reference genes. Each sample was analyzed in triplicate. All gene-specific primers were designed to amplify DNA fragments that encompass the junction of two neighboring exons.

### Measurement of absolute cDNA quantity

The absolute quantity of cDNA produced from *Cdh1*, *Cdh2*, and *Cdh3* mRNAs in mouse RPE was measured as previously described for other genes [36, 42] with modifications. To obtain standard curves for quantification, cDNA fragments of *Cdh1*, *Cdh2*, and *Cdh3* were generated from mouse RPE and choroid RNA by RT-PCR using primers that amplify the region including the segment to be quantified by RT-qPCR (S1 Table). Primer pairs for cDNA quantification were located at each side of exon-exon borders in respective cDNAs, and they were designed in the non-homologous region to avoid cross-reacting with other cadherin cDNAs. The PCR products were fractionated in agarose gels, purified using QIAquick Gel Extraction Kit (28704, Qiagen), and their DNA concentration was measured using NanoDrop Spectrophotometer (Thermo Fisher Scientific). To obtain the molecular complexity similar to the samples, mouse spleen cDNA was chosen to dilute the gel-purified DNA fragments for standard curves because the expression of these three cadherins in the spleen was barely detectable and the lowest among a variety of tissues tested. The purified DNA fragments were diluted into mouse spleen cDNA in a wide range of quantities and tested to determine the best range to generate standard curves that covered threshold cycle (Ct) values of the samples to be analyzed. As a result, the range of DNA fragments from 1 attomole (amole) to 0.1 zeptomole (zmole) was selected for standard curves. Sample cDNAs of mouse RPE were synthesized using 200 ng of total RNA in 20 μl reaction solution, along with mouse spleen cDNA, and all cDNAs were diluted by 20-fold for analyses in triplicate. The final cDNA quantity was calculated for 200 ng of total RNA for each sample by taking into account the process in which 1.5 μl of the 20-fold diluted cDNAs were used for real-time PCR reactions.

The absolute quantity of cDNA generated from *CDH1*, *CDH2*, and *CDH3* mRNAs in two types of human RPE (RPE *in situ* and M1 RPE primary cells) was measured in the same manner as described above for mouse RPE with minor modifications. To make standard curves, cDNA fragments of *CDH1*, *CDH2*, and *CDH3* were amplified from total RNA of small intestine, heart, and M1 cells, respectively by RT-PCR using primers that amplify the region containing the segment to be quantified by RT-qPCR (S1 Table). Primer pairs for cDNA quantification were made with the same design as described above for mouse genes. To dilute the gel-purified DNA fragments for standard curves, human retina, thymus, and retina cDNAs were used for *CDH1*, *CDH2*, and *CDH3*, respectively because these tissues barely express respective genes. The range of DNA fragments from 1 amole to 0.1 zmole was used for standard curves. Sample cDNAs were synthesized from 200 ng of total RNA. Considering the dilution and the volume of cDNA used in real-time PCR, the final cDNA quantity was calculated for 200 ng of total RNA.

### Immunofluorescence

For RPE flat-mounts, mouse eyes were dissected at the equator, the cornea and lens were removed, and the retina was carefully peeled off. The remaining eyecups containing the RPE and choroid were immediately fixed in 4% paraformaldehyde (PFA) in 0.1 M phosphate buffer for 10 min at room temperature and transferred into PBS. The eyecups were dissected into quarters by four radial cuts from the periphery toward the optic disc, and blocked in 0.25% Triton X-100 in Tris-buffered saline (TBST) with 10% normal horse serum (Z0610, Vector Laboratories, Burlingame, CA) and 1% bovine serum albumin (BSA; A9647, MilliporeSigma) at room temperature for 1 h. The RPE/choroid flat-mounts were incubated with a primary antibody in TBST containing 3% normal horse serum and 1% BSA at 4°C overnight with gentle shaking. After washing with TBS for 10 min three times at room temperature, the flat-mounts were incubated with appropriate secondary antibodies in TBST containing 1% BSA for 30 min at room temperature followed by washing with TBS for 10 min three times. The nuclei were stained with 4’,6-diamidino-2’-phenylindole dihydrochloride (DAPI, 10236276001, Roche, Indianapolis, IN) for 10 min at room temperature. The flat-mounts were washed with TBS, mounted in Fluorescent Mounting Medium (S3023, Dako, Carpinteria, CA), and images were acquired using an LSM 510 laser scanning confocal microscope (Carl Zeiss, Thornwood, NY). Primary antibodies used were anti-ZO-1 (dilution at 1:200; 402200, rabbit polyclonal, Invitrogen), anti-P-cadherin (1:200; AF761, goat polyclonal, R&D Systems, Minneapolis, MN), and anti-β-catenin (1:200; NBP1-32239, rabbit polyclonal, Novus, Littleton, CO). Secondary antibodies were anti-rabbit or anti-goat IgG conjugated with Alexa Fluor 488, 549, or 647 (1:500; Invitrogen). For staining F-actin (filamentous actin), the flat-mounts were incubated with CytoPainter Phalloidin-Fluor 555 Reagent (1:1000; ab176756, Abcam, Cambridge, MA) in PBS with 1% BSA for 30 min at room temperature.

To analyze the localization of P-cadherin and β-catenin, immunofluorescence of retinal sections was performed following the previously published protocols [43]. Briefly, mouse eyes were fixed in ice-cold 4% PFA in 0.1 M phosphate buffer pH 7.4 for 1 h, washed in 0.3% Triton X-100 in PBS (0.3% PBST), cryoprotected in an increasing gradient of sucrose (10%, 20%, and 30%) in PBS at room temperature for 1 h at each concentration, embedded in Tissue-Tek OCT (Ted Pella, Redding, CA), and snap frozen on dry ice in isopentane. Eye sections were cut at 10 μm on a cryostat, and incubated with the same primary and secondary antibodies as used for RPE flat-mounts, and examined in the same manner using an LSM 510 confocal microscope (Carl Zeiss) as described above.

### Western blot analysis

Protein lysates of mouse RPE were prepared according to the previously described method [44]. Mouse eyes were dissected in the same manner as described above for RPE flat-mounts. The eyecups were cut into 4 small petals and incubated in RIPA lysis buffer with protease inhibitor cocktail EDTA-free (R0278, MilliporeSigma) for 40 min on ice. After the insoluble fractions were removed by centrifugation at 14,000 rpm for 15 min at 4°C, the supernatants were collected, and protein concentration was determined using a BCA protein assay kit (Pierce, Thermo Fisher Scientific). The same amounts of proteins (25 μg) for each sample were subjected to 4–12% sodium dodecyl sulfate-polyacrylamide gel electrophoresis (SDS-PAGE) and transferred onto nitrocellulose membranes. The membranes were incubated for 1 h at room temperature with a primary antibody in TBS containing 0.05% Tween 20 (pH 7.4) in the presence of 5% nonfat dry milk. After washing the membranes in TBS containing 0.05% Tween 20, secondary antibody reactions were performed with an appropriate source of antibody conjugated with horseradish peroxidase. The signals were detected with an enhanced chemiluminescence (ECL) detection kit (RPN2232, GE Healthcare Life Science, Marlborough, MA) using an ImageQuant LAS 4000 scanner (GE Healthcare Life Science). The intensity of each band was quantified using the ImageJ software (http://rsb.info.nih.gov/ij/; National Institutes of Health, Bethesda, MD). Primary antibodies used were anti-P-cadherin (1:2000; AF761, R&D systems), anti-β-catenin (1:2000; NBP1-32239, Novus), anti-Snail (1:1000; 3895, mouse monoclonal, Cell Signaling, Danvers, MA), and anti-vimentin (1:3000; 5741, rabbit monoclonal, Cell Signaling). For internal control, anti-β-actin antibody (1:100000; A3854, mouse monoclonal, MilliporeSigma) was used.

### Statistical analysis

Unpaired Student’s t-test was used for statistical analysis. P value less than 0.05 was considered as statistically significant difference (*P* < 0.05).

## Results

### RNA extraction from mouse RPE and choroid individually

First, we wanted to establish a method for obtaining RNA separately from mouse RPE and choroid. In most previous studies analyzing mouse RPE gene expression, RNA was extracted from the mixed RPE/choroid due to technical difficulties. To resolve this issue, Wang et al. reported an RNA extraction method for mouse RPE without the choroid [40]. We modified Wang’s method to individually purify RNA from both mouse RPE and choroid. RPE cells were released from the RPE/choroid/sclera eyecup in RNAprotect reagent, and the remaining choroid/sclera was transferred to a new tube, which allowed RNA extraction from RPE cell pellets and the choroid/sclera in separate tubes (see the details in the Materials and methods section). Trizol enabled complete lysis of the choroid/sclera, which could not be achieved by the RNeasy lysis buffer, and RNeasy columns enabled to remove melanin pigments that inhibit downstream enzyme reactions.

**Fig 1:**
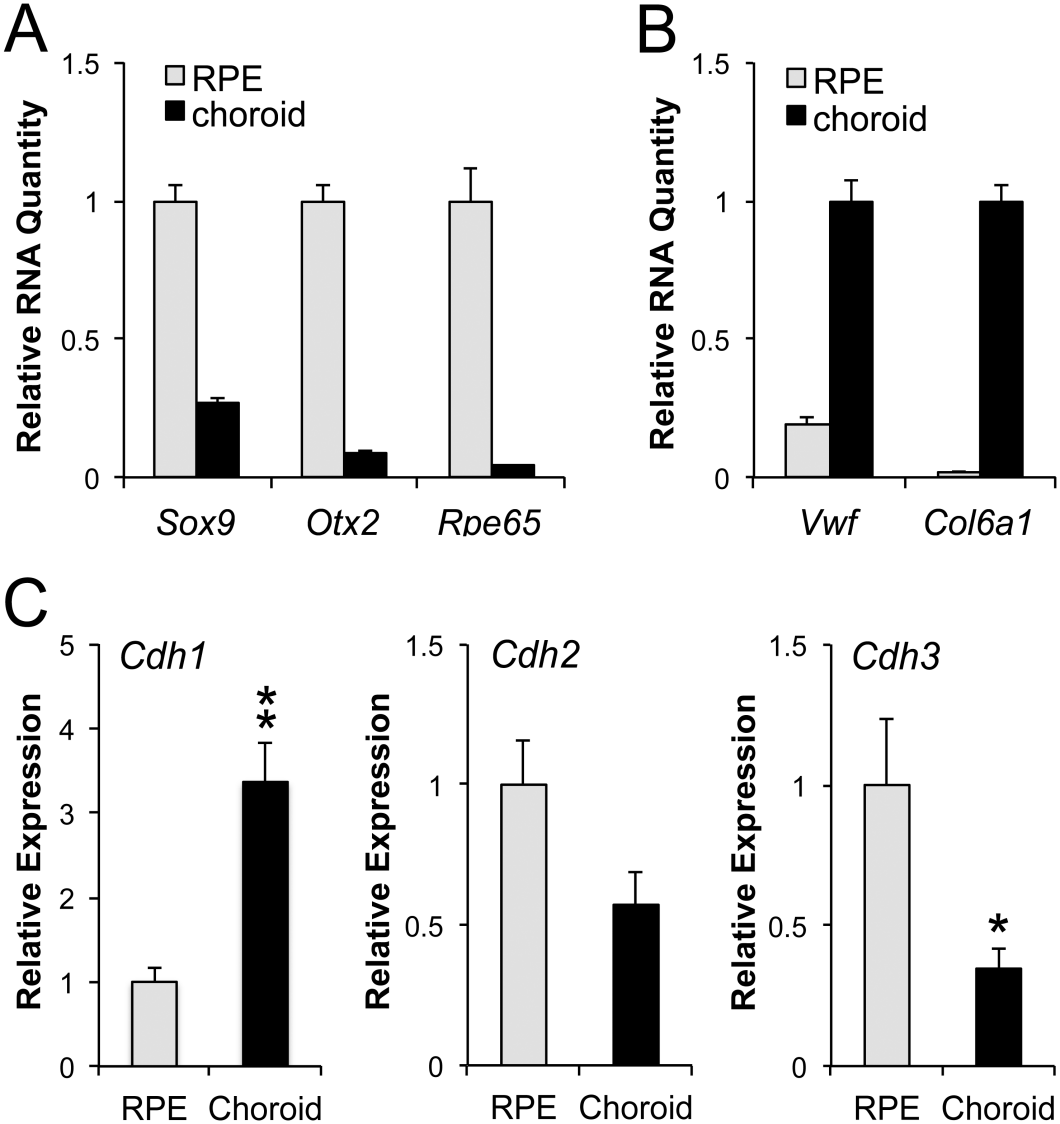
Cadherin subtypes show distinct preferential expression patterns in mouse RPE and choroid. (A) A method for extracting RNA individually from mouse RPE and choroid was established, and RNA samples were tested for cross-contamination. The expression of RPE markers (*Sox9*, *Otx2*, and *Rpe65*) in three biological replicates was analyzed by RT-qPCR in triplicate using *Gapdh*, *Hprt*, and *Actb* as reference genes. Relative RNA quantity was calculated as a ratio to the expression level in mouse RPE samples. The values represent the means and SEM (bar). (B) The same RNA samples were tested for cross-contamination using choroid markers (*Vwf* and *Col6a1*) by RT-qPCR in the same manner as in A. Relative RNA quantity was calculated as a ratio to the expression level in mouse choroid samples. The values represent the means and SEM (bar). (C) Total RNA from mouse RPE and choroid was prepared individually using the newly established method, and the mRNA expression of three cadherins was tested. RT-qPCR analysis was performed for *Cdh1* (gene for E-cadherin), *Cdh2* (N-cadherin), and *Cdh3* (P-cadherin) in the same manner as described in A. Relative expression was calculated as a ratio to the expression level in mouse RPE. The values represent the means and SEM (bar). Statistical significance is shown by * (p < 0.05) and ** (p < 0.01).

To test cross-contamination of RNA from mouse RPE and choroid/sclera, we analyzed the expression of RPE markers (*Sox9*, *Otx2*, and *Rpe65*) and choroid markers (*Vwf* and *Col6a1*) using RT-qPCR. The mRNA levels of *Sox9*, *Otx2*, and *Rpe65* in the choroid samples were 27%, 8%, and 4% of those in the RPE samples, respectively (Fig 1A). The result of *Sox9* seemed to reflect its expression in choroidal melanocytes. The mRNA levels of *Vwf* and *Col6a1* in the RPE samples were 19% and 2% of those in the choroid samples, respectively (Fig 1B). *Vwf* encodes von Willebrand factor that is expressed in platelets and vascular endothelial cells [45], which are abundantly present in the choroid, particularly beneath the Bruch’s membrane, leading to higher contamination in the RPE samples. *Col6a1* encodes collagen type VI alpha 1 chain, an extracellular matrix protein expressed in fibroblasts and adipocytes [46], showing no significant contamination in the RPE samples. The results showed that RPE and choroid/sclera RNAs were slightly contaminated with each other, but not to a substantial degree, suggesting that our method is useful for analyzing gene expression in mouse RPE and choroid individually.

### E-, N-, and P-cadherins show distinct preferential expression patterns in the RPE and choroid

Using our RNA extraction method described above, we prepared total RNA from mouse RPE and choroid separately in 3 biological replicates and analyzed the mRNA expression of the three cadherins by RT-qPCR. The mRNA level of *Cdh1* was significantly higher in the choroid than in the RPE by 3.4-fold (*P* = 0.0086) (Fig 1C). In contrast, the mRNA level of *Cdh3* was significantly higher in the RPE than in the choroid by 2.9-fold (*P* = 0.048). The mRNA level of *Cdh2* also showed a trend to be higher in the RPE than in the choroid, but the difference was not significant (*P* = 0.086). These results are suggestive of P-cadherin as a major cadherin in mouse RPE *in situ*. However, since the specificity and efficiency of gene-specific primers are different for each gene, we wanted to more quantitatively compare the expression levels across different cadherin mRNAs.

### P-cadherin is the dominant cadherin in normal mouse RPE *in situ*

**Fig 2:**
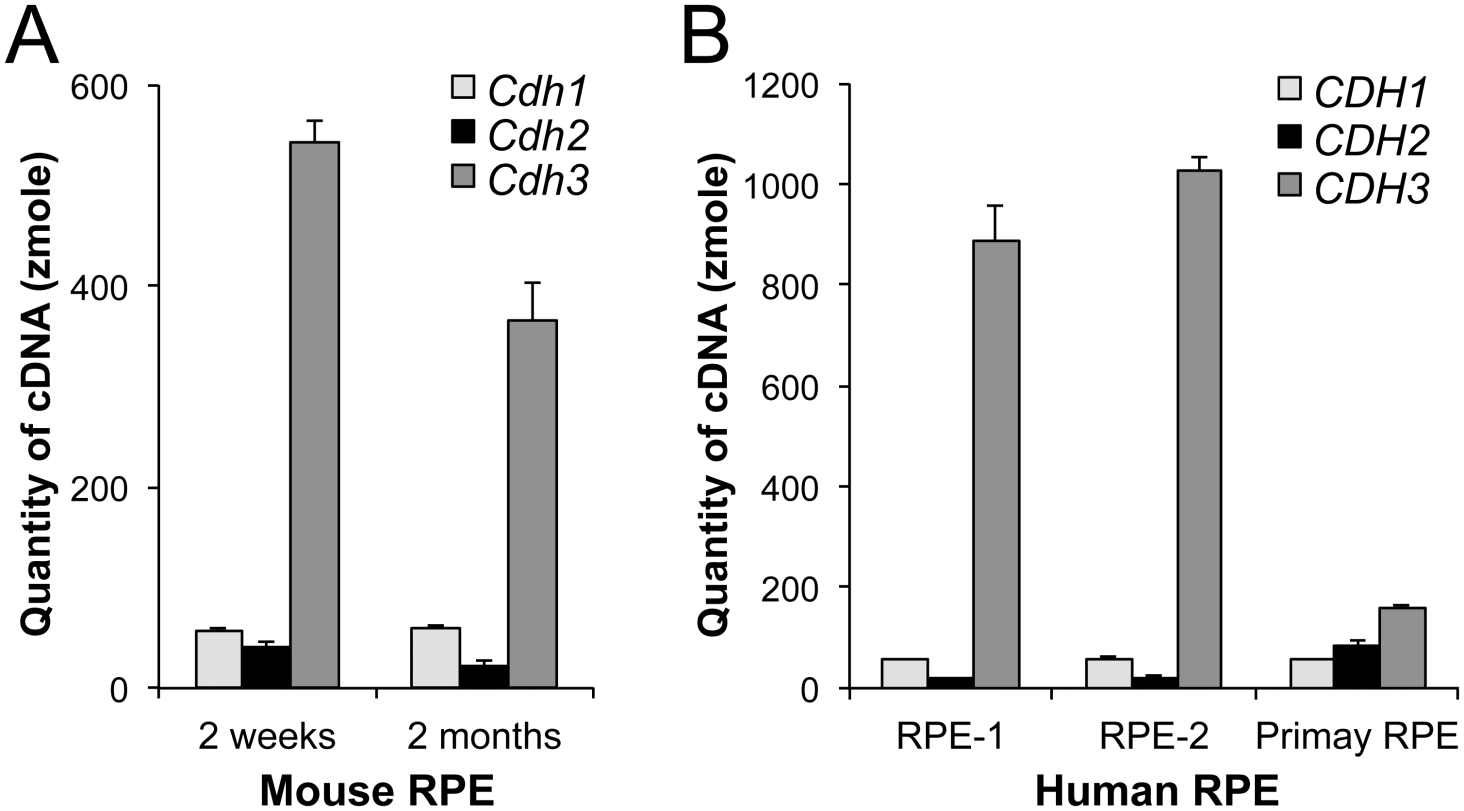
P-cadherin is the dominant cadherin in mouse and human RPE *in situ*. (A) Absolute quantification of cDNA to assess the mRNA quantity of *Cdh1*, *Cdh2*, and *Cdh3* in mouse RPE *in situ*. Total RNA was prepared from the RPE of 2 week-old and 2 month-old mice, and RT-qPCR was performed, along with gel-purified PCR products to create standard curves ranging from 1 attomole (amole) to 0.1 zeptomole (zmole). Based on Ct values of the standard curves, the quantity of cDNA for each gene was calculated for 200 ng total RNA used for cDNA synthesis. Three biological replicates were analyzed in triplicate for each sample. The values represent the means and SEM (bar). (B) Absolute quantification of cDNA to assess the mRNA quantity of *CDH1*, *CDH2*, and *CDH3* in human RPE. Total RNA was prepared from the RPE of two donor eyes (RPE-1 and RPE-2) and human RPE primary cells (M1), and RT-qPCR was performed in triplicate in the same manner as described in A, along with gel-purified PCR products to create standard curves. Based on Ct values, the quantity of cDNA for each gene was calculated for 200 ng total RNA. The values represent the means and SEM (bar).

We quantified the absolute amount of cDNA generated from mRNA of the three classical cadherins. Total RNA was prepared from 2 week-old and 2 month-old mouse RPE using our newly established RNA extraction method described above. Based on Ct values of the standard curves, the amount of cDNA produced from 200 ng total RNA was calculated as 57.4, 40.4, and 542 zmole at 2 week-old and 59.7, 22.4, and 365 zmole at 2 month-old for *Cdh1*, *Cdh2*, and *Cdh3*, respectively (Fig 2A). As control for the quantity and quality of RNA, we confirmed that the two RPE samples had the equivalent level of *Gapdh* expression. Assuming that the efficiency of RT reaction and PCR is the same for all samples and genes, we compared each cadherin mRNA expression directly based on the calculated cDNA quantity. These results show that *Cdh3* is the dominant cadherin expressed in mouse RPE *in situ* at the mRNA level, with *Cdh3* accounting for 82~85% of the total of the three cadherin expression.

### P-cadherin is the highly dominant cadherin in human RPE *in situ*, but not in cultured RPE cells

Next, we tested whether P-cadherin was also dominant in RPE cells of different species. Since RNA samples of human RPE, two directly extracted from donor eyes (RPE-1 and RPE-2) and one from RPE primary cells (M1) with cobble-stone-like morphology, were already available [36, 37], we analyzed these samples using the same quantification procedures as used for mouse RPE. Based on Ct values of the standard curves, the amount of cDNA produced from 200 ng total RNA was calculated as 55.7, 18.2, and 886 zmole in RPE-1, 57.9, 20.7, and 1030 zmole in RPE-2, and 57.0, 81.5, and 161 zmole in M1 for *CDH1*, *CDH2*, and *CDH3*, respectively (Fig 2B). As control for the quantity and quality of RNA, we confirmed that these RPE samples had the similar level of *GAPDH* and *HPRT* expression. As in the case of mouse RPE, these results clearly show that *CDH3* is the vastly dominant cadherin expressed in human RPE *in situ* at the mRNA level, with *CDH3* accounting for 92~93% of the total of the three cadherin expression. In contrast, primary RPE cells, even though they had the typical cobblestone-like morphology of well-differentiated RPE, did not show such a dominance of *CDH3* expression, and instead expressed *CDH2* at the higher level than RPE *in situ* (*P* = 0.017). These results confirm the dominance of P-cadherin expression as general characteristics of RPE cells *in vivo* and suggest the need for caution in interpreting results from cultured RPE cells.

### P-cadherin is co-localized with ZO-1, β-catenin, and F-actin at the cell-cell border of mouse RPE *in vivo*

**Fig 3:**
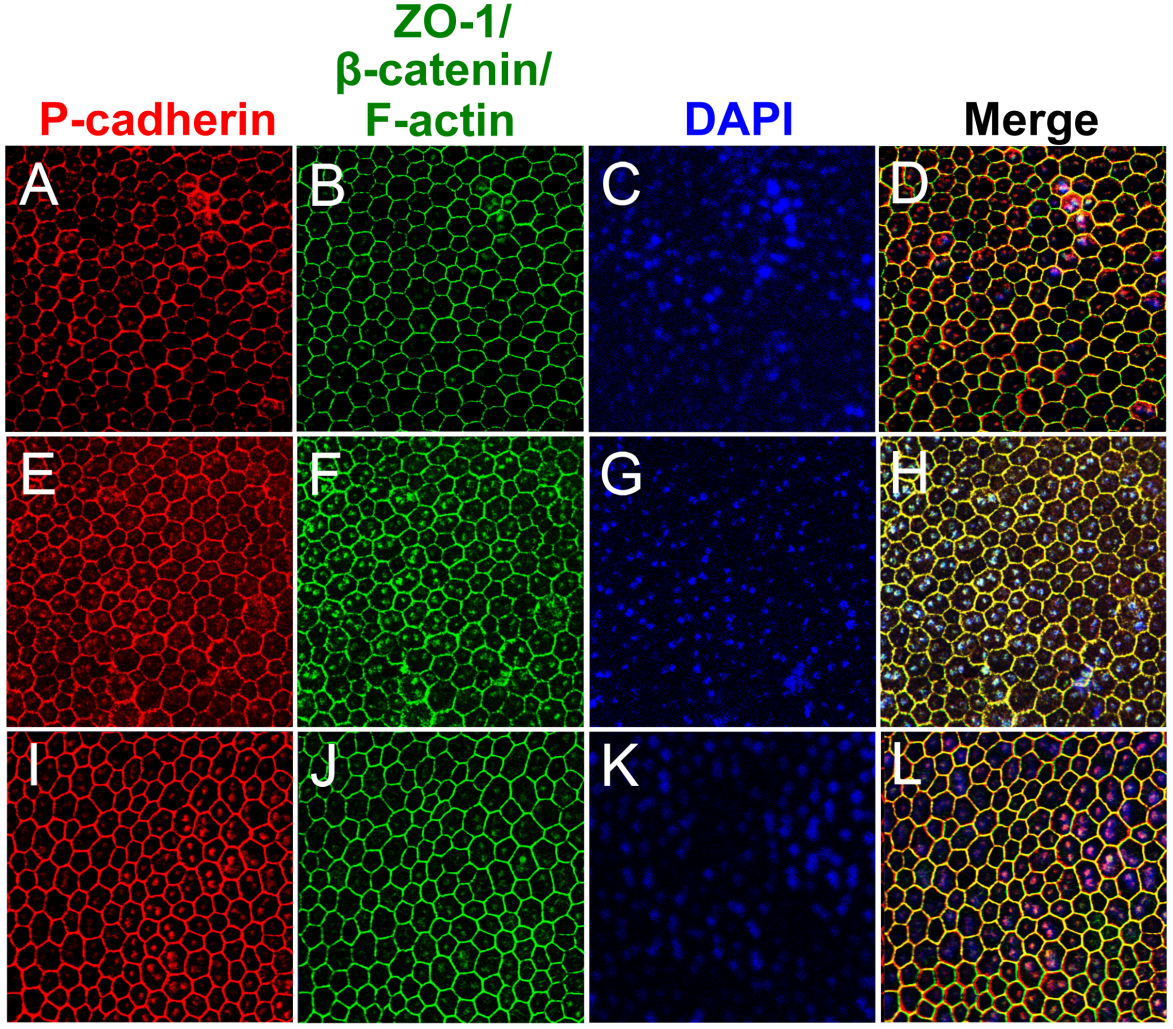
P-cadherin is co-localized with other junctional proteins at the RPE cell border in mice. Immunofluorescence of mouse RPE flat-mounts. Double staining: P-cadherin (red; A, E, I) and either ZO-1 (green; B), β-catenin (green; F), or F-actin (green; J), with nuclear stain by DAPI (blue; C, G, K). Merged images (D, H, L) show the co-localization of P-cadherin with ZO-1 (tight junction), β-catenin (adherens junction), and F-actin (adherens junction) at the cell-cell border.

To assess P-cadherin expression at the protein level, we used immunofluorescence of mouse RPE flat-mounts. We performed double staining for P-cadherin and either of the proteins that are localized at the cell-cell border, ZO-1 (tight junction protein 1, TJP1), β-catenin (a component of adherens junctions), or F-actin (associated with adherens junctions). We obtained a strong and clear signal of P-cadherin at the cell-cell border of mouse RPE *in situ*, and confirmed that P-cadherin was co-localized with all of the three proteins analyzed (Fig 3). This anti-P-cadherin antibody is described as being highly specific to mouse P-cadherin, with less than 0.3% cross-reactivity with recombinant mouse E-cadherin by sandwich immunoassays (AF761, R&D Systems), and has been used to study mouse epidermis and hair follicles [47, 48]. We also performed immunofluorescence for both E- and N-cadherins on mouse RPE flat-mounts with at least two different antibodies that worked for culture cells, but could not obtain a clear signal. These results confirm that P-cadherin protein is indeed expressed at the cell-cell border, and co-localized with other proteins that are known to compose cell junctions.

### Oxidative stress disrupts P-cadherin localization at the cell border

Based on the reported findings that oxidative stress disrupts cell junctions in cultured RPE cells [4, 5, 7, 49], we first tested the effect of oxidative stress on the localization of P-cadherin in mouse RPE *in vivo*. Sodium iodate (NaIO_3_), an oxidative stress inducer, was injected into mice via tail vein on Day 0, and immunofluorescence of mouse RPE flat-mounts was performed on Days 1, 2, 3, and 5. After oxidative stress, P-cadherin was no longer detected exclusively at the cell membrane, but instead its diffuse labeling was detected in the cytoplasm, suggesting that oxidative stress may cause P-cadherin dissociation from the adherens junctions. This diffuse cytoplasmic staining was observed most prominently on Days 2 and 3, followed by gradual decrease (S1 Fig). In contrast, the localization of ZO-1 at the cell-cell border was less disturbed by oxidative stress in the experimental conditions used. These results indicate that NaIO_3_-induced oxidative stress, even at a low dose, causes significant changes inside RPE cells at the molecular and structural levels, although RPE morphology looks grossly normal.

### Oxidative stress-induced dislocation of adherens junction proteins results in translocation of β-catenin to the nucleus

**Fig 4:**
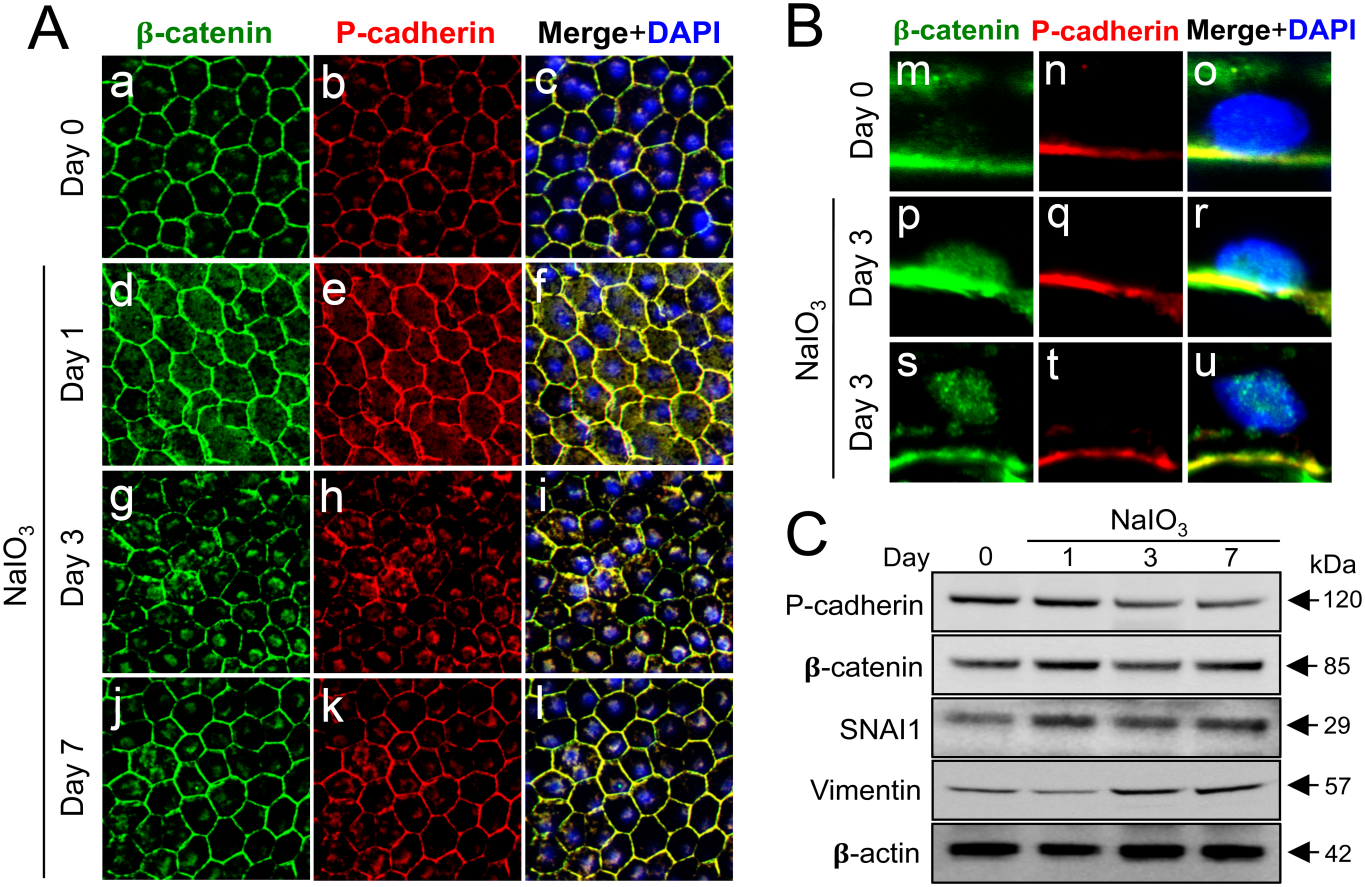
Oxidative stress-induced dissociation of adherens junctions results in nuclear translocation of β-catenin and an increase of EMT-related factors in mouse RPE. (A) Immunofluorescence of mouse RPE flat-mounts. Mice were injected with NaIO_3_ (15 mg/kg body weight) on Day 0, and the localization of β-catenin (green) and P-cadherin (red) was analyzed along with nuclear stain by DAPI (blue) on Days 0 (a-c), 1 (d-f), 3 (g-i) and 7 (j-l). Double staining: β-catenin (a, d, g, j), P-cadherin (b, e, h, k), and merged images with DAPI (c, f, i, l). The localization of β-catenin and P-cadherin at the cell-cell border was significantly disrupted, and instead prominently detected on/in the nucleus on Day 3. (B) Immunofluorescence of mouse retinal sections with a focus on the RPE nuclei. Mice were injected with NaIO_3_ (15 mg/kg body weight) on Day 0, and the localization of β-catenin (green) and P-cadherin (red) was analyzed along with nuclear stain by DAPI (blue) on Days 0 (m-o) and 3 (two representative nuclei; p-r and s-u). Double staining: β-catenin (m, p, s), P-cadherin (n, q, t), and merged images with DAPI (o, r, u). On Day 3, β-catenin was detected in the nuclei of mouse RPE. (C) Western blot analyses of mouse RPE proteins. Mice were injected with NaIO_3_ (15 mg/kg body weight) on Day 0, and RPE protein lysates were prepared on Days 0, 1, 3, and 7. The protein levels were analyzed using Western blotting with antibodies against P-cadherin, β-catenin, SNAI1 (Snail), vimentin, and control β-actin. The protein levels of β-catenin and SNAI1 increased similarly on Day 1 following oxidative stress.

After observing that oxidative stress resulted in a dramatic change in the distribution of P-cadherin in mouse RPE, we next investigated the localization of β-catenin, another component of adherens junctions as well as a key factor of the Wnt/β-catenin signaling pathway. NaIO_3_ was injected into mice in the same manner on Day 0, and double staining of RPE flat-mounts for P-cadherin and β-catenin was performed on Days 1, 3, and 7 (Fig 4A). Adherens junctions marked by P-cadherin and β-catenin became wider and more diffuse at the cell-cell border, and a portion of these proteins was also detected in the cytoplasm on Day 1 (Fig 4Ad–4Af). The most notable change was observed on Day 3 in that staining of both P-cadherin and β-catenin at the cell membrane was significantly weaker but instead became prominent around or on/in the nucleus (Fig 4Ag–4Ai). By Day 7, however, the localization of these adherens junction proteins mostly returned to the normal state on the cell membrane (Fig 4Aj–4Al). These results raised a critical question where P-cadherin and β-catenin were located on Day 3, outside or inside the nucleus. To answer this question, we performed immunofluorescence of mouse retinal sections on Day 3 after NaIO_3_ injection (Fig 4B). The nuclei stained by DAPI were mostly devoid of β-catenin and completely free of P-cadherin on Day 0 (Fig 4Bm–4Bo). In contrast, β-catenin was strongly and clearly detected inside the nuclei on Day 3, with P-cadherin staying negative in the nuclei, indicating that β-catenin was translocated to the nucleus (Fig 4Bp–4Bu). These results suggested activation of the canonical Wnt/β-catenin signaling pathway.

To obtain further evidence for the activation of the Wnt/β-catenin pathway, we analyzed the protein level using Western blotting with mouse RPE protein lysates on Days 1, 3, and 7 following NaIO_3_ injection. Indeed, β-catenin was increased by approximately 2-fold on Day 1, suggesting that β-catenin protein was stabilized (Fig 4C). We confirmed this result by repeating the same experiment using Western blotting. These results support that oxidative stress indeed leads to activation of the canonical Wnt/β-catenin pathway through the dissociation of adherens junctions and a subsequent release and stabilization of β-catenin.

### Oxidative stress induces EMT-related factors in mouse RPE

The next question was how mouse RPE cells were affected as the consequence of this activation of the Wnt/β-catenin pathway induced by oxidative stress. Based on the literatures reporting that activation of β-catenin can promote EMT in other cell types [12, 50, 51], we hypothesized that mouse RPE might also acquire EMT-like features by the activated Wnt/β-catenin pathway following dissociation of adherens junctions. Therefore, we analyzed three proteins possibly related to EMT by Western blotting (Fig 4C). The protein level of SNAI1, one of the well-known EMT transcription factors, increased on Day 1 in a similar manner to that of β-catenin, whereas the level of vimentin, a mesenchymal marker, gradually increased with time. Conversely, the level of P-cadherin, an epithelial marker, gradually decreased with passing days, suggesting that cell-cell contacts by adherens junctions may be weakening, which is consistent with EMT-like change.

## Discussion

RPE cells can dedifferentiate and lose their fully matured state through an EMT-like process in response to various stressors, including oxidative stress and Ca^2+^ removal, that result in the loss of cell-cell contact [4, 5, 7–9]. Recently, RPE EMT has drawn increasing interests, not only related to PVR [14, 16, 52] but also due to its potential relevance to the pathophysiology of dry AMD [10]. To trigger EMT, the loss of cell-cell contact seems critical as an initial event, as suggested by the findings that TGFβ, a known EMT inducer, could not initiate EMT in the central region of cultured porcine RPE sheets where cell-cell junctions were well maintained [9]. Adherens junctions regulate cell-cell contacts with neighboring cells and thereby maintain epithelial cell morphology, structure, and polarity [18–23]. Cadherins are key components of adherens junctions, but the cadherin subtype(s) playing a major role in forming and maintaining adherens junctions in RPE cells has been ambiguous. This is because most previous studies have analyzed cultured RPE cells, either ARPE19 cell line or RPE primary cells, with a limited number of *in vivo* studies, and because the methods used were not suitable for quantitative comparison [4, 5, 7, 8, 25, 26, 28, 49, 53–55]. In addition, most of these studies have focused on E- and/or N-cadherin, but not P-cadherin. Therefore, we wanted to clarify relative contribution of specific cadherin subtypes to formation of adherens junctions in RPE cells *in vivo*.

Our results show that P-cadherin mRNA is dominantly expressed in both human and mouse RPE *in situ* compared with E- and N-cadherins. We designed all cadherin primers for PCR based on the known RefSeq mRNA sequence for a preproprotein that undergoes proteolytic processing to generate a mature protein (National Center for Biotechnology Information, NCBI). These primers produce PCR fragments encompassing an exon-exon border in respective cDNAs in the region that is not homologous across the three cadherins, and are also included in all splice variants currently listed in the GenBank (NCBI). Using RNA from various tissues with different cadherin expression patterns, we confirmed that our primers do not cross-react with other cadherin cDNAs, supporting the validity of our findings.

The degree of dominance of P-cadherin expression in mature RPE *in vivo* was surprising; however, there are reports, although a limited number, that described P-cadherin expression in RPE cells of various mammalian species. Transcriptome analyses of native human fetal and adult RPE identified both *CDH1* and *CDH3* among 154 RPE signature genes [56]. However, since microarray data cannot be used to compare the expression of different genes, it was unclear which was the major cadherin in native RPE cells. Studies with human RPE primary cells reported that while N-cadherin was expressed in both early (2–3 days) and late (8 weeks) confluence as well as in both epithelioid and fusiform cells, E- and P-cadherins were detected only in late confluence and epithelioid cells [53]. Using Western blot analyses, these authors showed that both E- and P-cadherins were present in RPE cell extracts prepared from human donor eyes [53]. In mouse eye development, *Cdh3* mRNA was not detectable at E10.5 in the outer layer (future RPE) of the optic cup by *in situ* hybridization, but became highly expressed from E12 onward [28]. For bovine RPE *in situ*, the presence of P-cadherin protein was confirmed by Western blot analysis [57]. In cultured porcine RPE sheets, P-cadherin protein was abundantly present in RPE cells in the central region, where the cells maintained the cobblestone-like morphology, but lost in migrating cells at the edge of RPE sheets, where such cells began to express the EMT markers vimentin and N-cadherin [9]. Although these studies are not for quantitative comparison, they support our results regarding P-cadherin expression.

Based on these reports and our results, P-cadherin indeed seems to be the major cadherin that forms adherens junctions and maintains the epithelial phenotype of RPE cells *in vivo*. The functional importance of P-cadherin in the RPE is strongly supported by genetic case studies describing that *CDH3* mutations are responsible for two rare autosomal recessive disorders: HJMD [29–32] and EEM syndrome [33]. HJMD patients show early-onset hair loss and severe retinal dystrophy, particularly at the RPE. EEM syndrome exhibits additional features such as split hand/foot malformation (ectrodactyly) with/without dental malformations in addition to HJMD characteristics. These phenotypic features of *CDH3* mutations seem to reflect the expression patterns and function of P-cadherin, which plays a role in epithelial outgrowth and formation during development, including those of hair follicles, limb buds, mammary gland, and RPE [19, 34, 47, 58, 59]. The reason why *CDH3* mutations exclusively affect RPE cells in the macula is unclear, and answers to this question may also provide mechanistic insights into other macular degeneration including AMD. Based on our results, we speculate that mutated P-cadherin proteins may form weakened adherens junctions, leading to the higher susceptibility to oxidative stress-induced impairment of the RPE integrity. The macular region likely faces stronger oxidative stress due to the higher density of retinal photoreceptors and the densest choroidal vasculature including choriocapillaris in the submacula [60–62]. However, further investigation is needed to answer this important question.

In contrast to human HJMD patients, P-cadherin-deficient mice are viable and fertile with no overt developmental abnormalities including ocular phenotype [34]. However, it is unclear how extensively the eyes of P-cadherin knockout mice have been analyzed at the histological and molecular levels. Even with normal mice, surprisingly few reports described P-cadherin in mouse RPE *in vivo*, and this scarcity of reported analyses of P-cadherin further motivated us to carry out our present studies. Besides the *in situ* hybridization analyses described above [28], we could find only one other report that analyzed P-cadherin in mouse RPE *in vivo* by searching in the PubMed (NCBI). Using immunofluorescence, this report described that albino RPE cells are irregularly shaped, and their adherens junctions distribute more widely and loosely at the cell membrane than those of pigmented RPE cells, suggesting that the lack of melanogenesis may impair RPE cell integrity in albino mice [35]. Interestingly, we observed somewhat similar wider and loose distribution of P-cadherin and β-catenin at the cell membrane after oxidative stress.

The surprisingly dominant expression of P-cadherin in RPE *in situ* at the mRNA level prompted us to test the expression at the protein level. We obtained a clear strong signal for P-cadherin by immunofluorescence of mouse RPE flat-mounts, which showed the co-localization of P-cadherin with β-catenin, F-actin, and ZO-1, proteins known to locate at the cell-cell border. In addition, in response to NaIO_3_-induced oxidative stress, P-cadherin quickly lost its tight localization at the cell-cell border and instead distributed more diffusely in the cytoplasm, suggesting that P-cadherin forms adherens junctions in normal conditions. These results are consistent with the findings that oxidative stress induced by hydrogen peroxide (H_2_O_2_) disrupts junctional integrity of human ARPE19 cells and porcine RPE primary cells, including the loss of β-catenin localization at the cell border and the leak through tight junctions [4, 7]. Light exposure, which induces oxidative stress in the retina, has also been reported to rapidly disrupt the localization of ZO-1, β-catenin, and N-cadherin at the cell-cell border of mouse RPE, leading to their redistribution to the cytoplasm [8]. In these studies, however, β-catenin and N-cadherin, but not P-cadherin, were analyzed as junctional proteins as in other previous studies using cultured RPE cells.

Following the observation of oxidative stress-induced dissociation of adherens junctions, an important question was whether dislocation of β-catenin resulted in activation of the canonical Wnt/β-catenin signaling pathway. Indeed, we found both an increase of β-catenin protein and its translocation to the nucleus, hallmarks of activation of the canonical Wnt/β-catenin pathway. Our results seem consistent with the findings previously reported in other cell systems in several aspects as summarized below. 1) Effect of oxidative stress on the canonical Wnt/β-catenin pathway. NIH3T3 or HEK293 cells treated with H_2_O_2_ showed an increase of β-catenin and activation of T-cell factor (TCF), a transcriptional activator with which β-catenin forms a complex to regulate its target genes [63]. This β-catenin activation by oxidative stress was independent of Wnt ligands and triggered by the dissociation of dishevelled (DVL), an intermediate component of Wnt/β-catenin signaling, from nucleoredoxin, a member of the thioredoxin family that interacts with DVL and keeps it in an inactive state [63, 64]. It has also been reported that 4-hydroxynonenal, a lipid peroxidation product, activated the Wnt/β-catenin pathway in a rat model of diabetic retinopathy [65]. 2) Intersection of two β-catenin pools. β-catenin is an essential component of both cadherin-based adherens junctions and the canonical Wnt/β-catenin signaling pathway, and a connection of these two β-catenin pools was suggested and later demonstrated [66–68]. In A431 epidermoid carcinoma cells exposed to lysophosphatidic acid that forces rapid dissociation of adherens junctions, cadherin-bound β-catenin was internalized together with E-cadherin, accumulated at the perinuclear endocytic recycling compartment, and translocated into the nucleus, suggesting that dissociation of adherens junctions can affect β-catenin levels available for the Wnt/β-catenin pathway [67]. 3) The Wnt/β-catenin pathway and EMT. β-catenin in the nucleus binds to members of the TCF/LEF family of transcription factors to promote EMT [50, 51]. During mouse embryonic development, a stabilized form of β-catenin in epiblasts led to activation of Wnt/β-catenin target genes, and cells of the embryonic ectoderm exhibited a premature EMT as a consequence [51]. Snail, one of the key EMT transcription factors, has multiple intersections with the Wnt/β-catenin pathway. Activation of this pathway results in upregulation of Snail, and Snail protein stability and subcellular localization are regulated through phosphorylation by glycogen synthase kinase-3β (GSK-3β), an intermediate component of the Wnt/β-catenin pathway [69, 70]. In addition, Snail interacts with β-catenin and increases its transcriptional activity, indicating a positive feedback stimulation of the Wnt/β-catenin pathway by Snail [71]. Accordingly, although multiple pathways are involved in EMT, oxidative stress-induced EMT through activation of the Wnt/β-catenin pathway needs to be considered as one of the consequences of oxidative stress in RPE cells.

The findings reported so far suggest that cadherin subtype profiles are different between cultured RPE cells and mature RPE *in vivo*, and that P-cadherin be the major cadherin in the latter, but not in the former. However, this issue has not been clearly addressed thus far. In most epithelial cell types, E-cadherin is the major cadherin that is responsible for forming and maintaining their epithelial phenotype [18, 20, 23]. In contrast, it has been reported that RPE cells dominantly express and use N-cadherin to form adherens junctions [24–26]. However, it should be noted that these results were obtained from cultured RPE cells, such as ARPE19 human RPE cells and human RPE primary cells. Retrospectively, these results are not surprising because our quantification data show that M1 human RPE primary cells with the cobblestone-like appearance lost the dominant expression of *CDH3* and instead acquired the expression of *CDH2* at the level significantly higher than that in RPE *in situ*. In our previous studies, we also found that while M1 cells expressed RPE markers such as *MITF* and *OTX2*, they were quite different from *in situ* RPE cells with regard to the expression profile of MITF isoforms and the level of *RPE65* expression [36]. Therefore, this difference in cadherin expression profiles is another example to add to the accumulating evidence of the difference between cultured RPE cells and RPE *in situ*.

The role of typically epithelial cadherins, E- and P-cadherins, and mesenchymal cadherin, N-cadherin, has become recognized as being more complex than originally defined, with both overlapping and distinct functions [17, 19, 72]. Interestingly, studies of epidermal sheet formation and maintenance showed that the level of cadherin is more critical than the subtype [48]. This finding seems consistent with the observation that the outcomes of P-cadherin overexpression in breast cancer depend on the cellular context of E-cadherin. In cells in which E-cadherin is highly expressed and maintained at the cell-cell border, P-cadherin disrupts E-cadherin function and promotes invasion, whereas in cells without E-cadherin, P-cadherin promotes adhesion and suppresses invasion [19, 59, 73]. In the case of *in situ* RPE with the barely detectable level of E-cadherin, P-cadherin likely promotes adhesion by forming adherens junctions. However, the situation in cultured RPE cells is quite different. Without the dominant abundant expression of typically epithelial cadherin, E- or P-cadherin, it is likely that N-cadherin plays a role in forming adherens junctions in cultured RPE cells, although N-cadherin is usually associated with EMT in most of other epithelial cell types [12, 72].

## Conclusions

We have established the RNA extraction method to purify RNA from mouse RPE and choroid individually. Using this method, we identified P-cadherin as the highly dominant form of cadherin in mouse RPE *in situ*. P-cadherin was also dominantly expressed in human RPE *in situ*, but not in cultured RPE cells. In addition, we found that oxidative stress led to dislocation of adherens junction proteins, P-cadherin and β-catenin, from the cell membrane to cytoplasm, resulting in nuclear translocation of β-catenin, a sign of activation of the canonical Wnt/β-catenin signaling pathway, and ultimately EMT-like molecular changes as a consequence. Based on this study, we would like to suggest that the expression and function of cadherins, especially P-cadherin, in the RPE should be reevaluated.

## Supporting information

S1 Table Primer sequences. Sequences of all primers used in this study for expression analyses and generation of cDNA fragments for standard curves.
(XLSX)

S1 Fig Oxidative stress disrupts P-cadherin localization at the RPE cell junctions in mice. Immunofluorescence of mouse RPE flat-mounts. Mice were injected with a low dose of NaIO_3_ (15 mg/kg body weight) on Day 0, and the localization of P-cadherin protein was analyzed on Days 1 (A, B, C, D), 2 (E, F, G, H), 3 (I, J, K, L) and 5 (M, N, O, P). Double staining: P-cadherin (red; A, E, I, M) and ZO-1 (green; B, F, J, N), with nuclear stain by DAPI (blue; C, G, K, O) and merged images (D, H, L, P).
(TIF)
