# Supplementary figures and images for: Cadherins in the retinal pigment epithelium (RPE) revisited: P-cadherin is the highly dominant cadherin expressed in human and mouse RPE in vivo
Source: PLoS One. 2018 Jan 16;13(1):e0191279. doi: 10.1371/journal.pone.0191279 (PMC5770047; doi:10.1371/journal.pone.0191279)

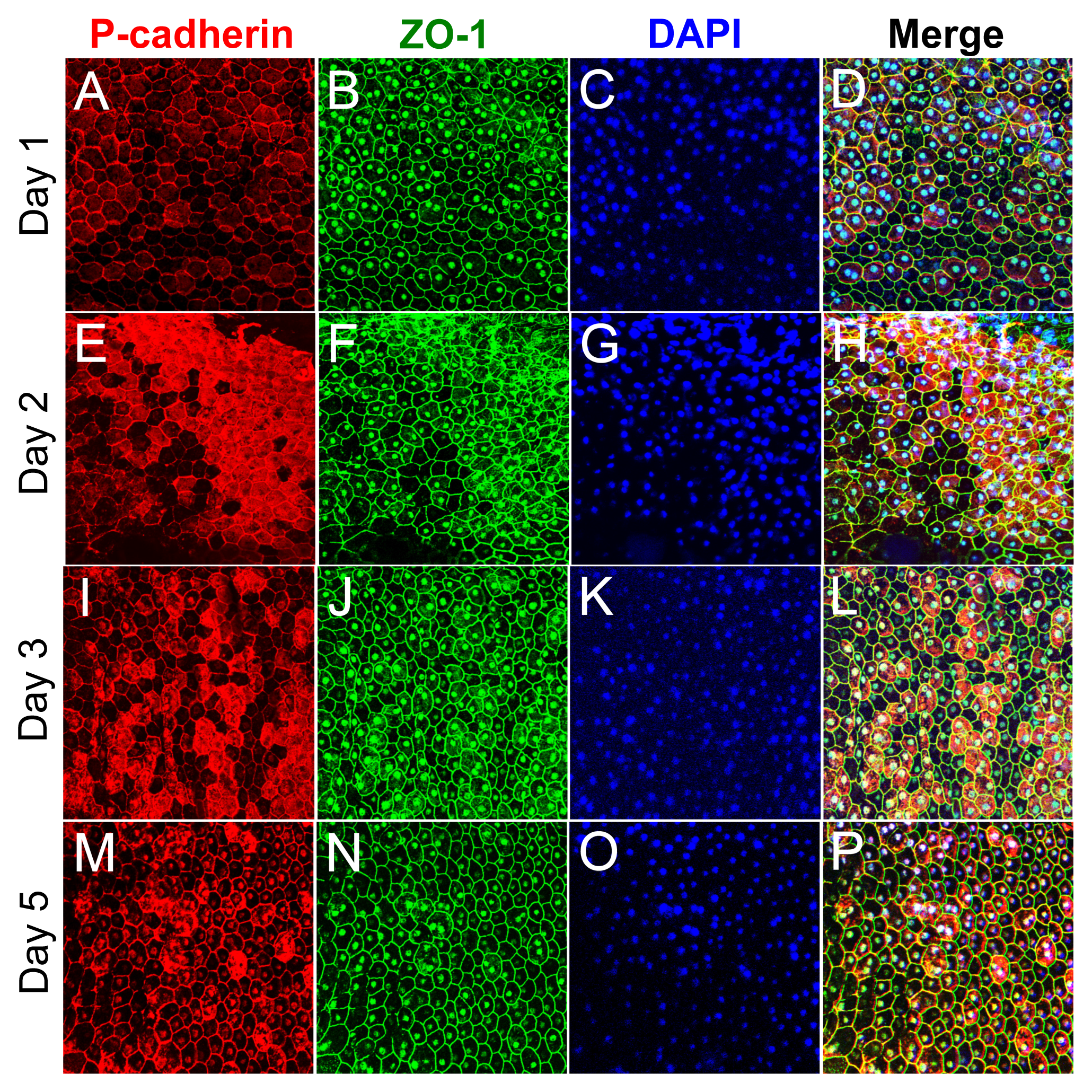

Supplement: S1 Fig — Immunofluorescence of mouse RPE flat-mounts. Mice were injected with a low dose of NaIO3 (15 mg/kg body weight) on Day 0, and the localization of P-cadherin protein was analyzed on Days 1 (A, B, C, D), 2 (E, F, G, H), 3 (I, J, K, L) and 5 (M, N, O, P). Double staining: P-cadherin (red; A, E, I, M) and ZO-1 (green; B, F, J, N), with nuclear stain by DAPI (blue; C, G, K, O) and merged images (D, H, L, P). (TIF) [file pone.0191279.s002.tif]
